# Promoting regular dental attendance in problem‐orientated dental attenders: A systematic review of potential interventions

**DOI:** 10.1111/joor.13244

**Published:** 2021-08-23

**Authors:** Charlotte C. Currie, Vera Araujo‐Soares, Simon J. Stone, Fiona Beyer, Justin Durham

**Affiliations:** ^1^ School of Dental Sciences Newcastle University Newcastle Upon Tyne UK; ^2^ Newcastle Upon Tyne Hospitals NHS Trust Newcastle Upon Tyne UK; ^3^ Faculty of Behavioural, Management and Social Sciences University of Twente Enschede The Netherlands; ^4^ Population Health Sciences Institute Newcastle University Newcastle Upon Tyne UK

**Keywords:** dental anxiety, dental care, dental health services, dental utilisation, systematic review

## Abstract

**Background:**

Problem‐orientated dental attenders account for around one‐third of the UK population, these being patients who do not seek regular dental care, instead only attending with dental pain. In order to develop intervention(s) to encourage regular dental attendance in these patients, any previous intervention development should be identified to aid idea generation or retrofitting of interventions.

**Objective:**

To identify previous interventions which have been developed targeted at problem‐orientated dental attenders to facilitate the development and co‐design of a new intervention.

**Methods:**

Eight electronic databases were searched for studies which included an intervention targeted at adult problem‐orientated or irregular dental attenders to encourage regular dental attendance. Data on the intervention design mapped to the theoretical domains framework were extracted, alongside effectiveness and patient views where available.

**Results:**

Three studies fitted the inclusion criteria for the review. Interventions identified were attendance at a dental anxiety clinic, and a large advertising campaign promoting a free dental update where members of the public could visit local dental practices to look around and meet the dentists. One study looked at the effect of policy change by introducing free dental check‐ups in Scotland. Interventions were poorly reported, with significant omissions in their description and a lack of clear identification of what composed the intervention.

**Conclusion:**

There are very few interventions developed targeted at problem‐orientated dental attendance, but important areas to consider in future intervention development include the following: dentist communication; dentist‐patient relationship; increasing the awareness of need; the effect of free dental check‐ups.

## Graphical abstract

A systematic review was carried out to identify previously developed interventions targeted at problemorientated dental attendance. Three studies were identified. Interventions included attendance at a dental anxiety clinic, a promotional campaign, and policy change introducing a free dental check‐up. Interventions were poorly reported but identified potential areas for future intervention development.

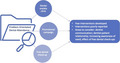

## 1 INTRODUCTION

Almost one‐third of the UK population do not seek regular dental care, instead only attending when suffering with acute dental pain or dental problems^1^ often suffering for a prolonged period of time beforehand.^2^ These problem‐orientated attenders can present to a range of healthcare professionals, including dentists,^3^ general medical practitioners^4^ and medical emergency departments^5^ often on a repeated basis.^3^ Seeking treatment from non‐dental providers often results in temporary treatment, such as a prescription for analgesics or potentially inappropriate antibiotics and advice to see a dentist, thus putting these patients into a cycle of repeat attendance. This attendance pattern is not exclusive to the UK with estimates of regular/preventive utilisation being 54% globally.^6^ Despite this, there is a scarcity of research developing and examining interventions to encourage regular over problem‐orientated dental attendance in all age groups. In addition, social inequalities are known to exist within regular/preventive dental care utilisation, and there is also a lack of research into interventions aiming to reduce this.^7^

Little is known about the healthcare seeking behaviour of problem‐orientated dental attenders, although it is acknowledged that a ‘web of causation’^8^ and social inequalities^9^ are likely to influence this attendance pattern. These complexities underpinning problem‐orientated dental attendance make designing and developing interventions challenging in this patient group. To successfully develop any new interventions, it is important to consider existing interventions which have already been designed and trialled to identify components which could be improved or incorporated (retrofitted) or discounted as ideas.^10^ To facilitate this, any previous interventions need to be mapped to their theoretical basis to facilitate the description of its mechanism of action and active ingredients. The Theoretical Domains Framework (TDF)^11^ is a framework aimed at aggregating under broader domains a multitude of behavioural theories and the constructs associated with these. Understanding what domain(s) an intervention is aimed at targeting allows the generation of evidence informed hypothesis on the basic mechanisms of action underlying the intervention. This, in turn, can link it to its active ingredients.

The aim of this systematic review was to investigate previous interventions or healthcare policy which have been developed for, and targeted at, problem‐orientated dental attenders to facilitate the development and co‐design of a new intervention. Where possible secondary aims were to establish the effectiveness and to consider views or opinions of any patients or healthcare providers on any existing interventions and healthcare policy identified.

## 2 MATERIALS AND METHODS

This systematic review was conducted with an *a priori* protocol, which was published online on PROSPERO^12^ and reported following PRISMA guidance.^13^

The criteria for considering studies for the systematic review were (PICOS):

- *Participants*: patients above the age of 18 years old of any gender who were problem‐orientated or irregular dental attenders. Studies of patients below 18 years old and of patients attending with chronic oro‐facial pain were excluded.
- *Interventions*: any form of intervention (brief or complex) or policy change that encouraged regular dental attendance instead of problem‐orientated attendance were included.
- *Comparators*/*Control*: No comparator was mandatorily required for inclusion. Where effectiveness was to be specifically considered the comparator or control group was set as patients not receiving the intervention, or for policy change the effect before and after.
- *Outcomes*: Primary outcomes were an increase in regular or preventive dental care visiting/utilisation or an increase in emergency attendance at a dentist instead of other healthcare providers.
- *Studies*: All peer‐reviewed English language studies of any design were included. Where effectiveness was to be specifically considered, only studies using a comparative design for interventions and controlled before‐after studies for policy change were included.

### 2.1 Search methods

Eight electronic databases were searched up to 1 April 2021 (Appendix S1): Medline via OVID; Embase via OVID; Scopus vis SciVerse; PsycINFO via OVID; Cochrane Central Register of Controlled Trials (CENTRAL); Cochrane Database of Systematic Reviews; Database of Abstracts of Reviews of Effects; NHS Economic Evaluation Database (NHS EED). The search strategy (Appendix S2) was primarily developed for Medline and then revised appropriately for each database to take into account the differences in controlled vocabulary and syntax rules. The references of eligible studies were also searched for further potential papers for inclusion. Grey literature was not included in the search.

### 2.2 Data collection and analysis

Eligible studies were selected by the first reviewer (CC) according to the inclusion and exclusion criteria based upon the study title and abstract (where available). If it was unclear whether a study should be included or not the full text was reviewed. A second reviewer (JD) reviewed the full text of all potential studies for inclusion, blinded to the journal title, institutions involved and the authors. Any disagreement regarding the inclusion of any study were resolved by discussion and inclusion of a third reviewer.

### 2.3 Data extraction and management

A standardised form was created in Microsoft Word (Microsoft Office Professional Plus 2016) and used to extract data from the included studies. The first reviewer (CC) extracted and entered the data onto the form. To ensure reliability, all of the extracted data were cross‐checked by the second reviewer (JD), again blinded to the study details as described above. Any disagreement was resolved as previously described. Data extracted included the following:

- Intervention/policy change design, type and details, classified where possible using the Theoretical Domains Framework (TDF)^11^
- Target population
- Outcome(s)
- Cost of intervention/policy change (if available)
- Patient and healthcare provider views (if available)
- Author's conclusion(s) and recommendation(s)
- Citation information

A risk of bias assessment was not considered necessary for studies included in the review as the outcome of the assessment would not have affected the decision on whether to develop ideas or components further. If multiple studies had been identified with appropriate study design and comparators to consider effectiveness in detail then the ROBINS‐I tool would have been used for risk of bias assessment.^14^

### 2.4 Data synthesis

Studies were tabulated to describe the intervention or policy change and to map to the TDF domains where possible. This provided a descriptive analysis to display ideas for further development. Due to substantial heterogeneity between the eligible studies effectiveness was summarised by study and integrated into a narrative synthesis of the main findings with a descriptive analysis only. Where patient or provider views and opinions were available these were considered alongside the intervention or policy change and integrated into a narrative synthesis.

## 3 RESULTS

**FIGURE 1:**
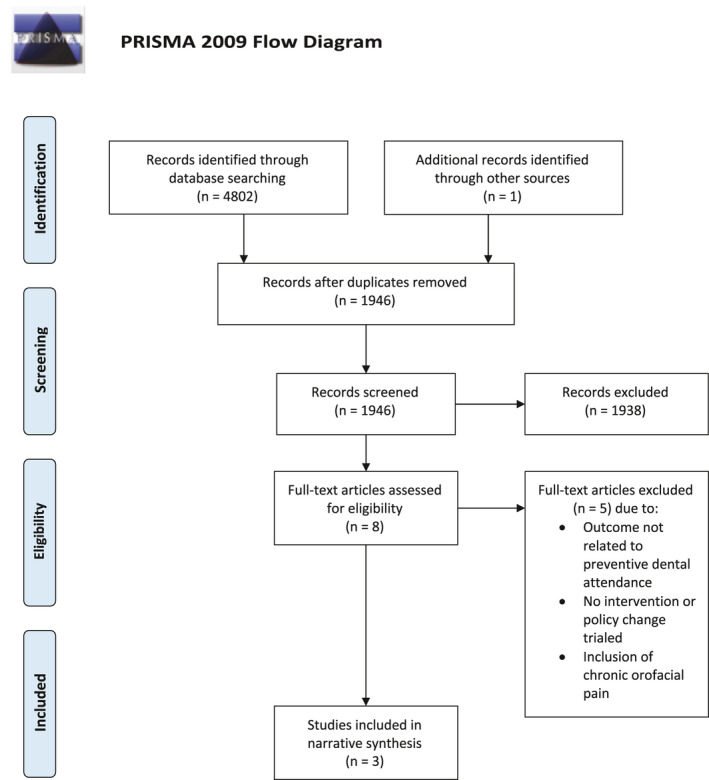
Prisma diagram

The search strategy initially identified 4803 articles (Figure 1), of which 2857 were identified as duplicates leaving 1946 studies for initial review. Fifty‐five papers were excluded as they were non‐English, however from their English title or abstract did not appear relevant to the review. Following screening for inclusion and exclusion criteria, eight studies were reviewed as a full text, with three being included in the final review.^15^, ^16^, ^17^ A list of the 5 excluded studies^18^, ^19^, ^20^, ^21^, ^22^ is given in Appendix S3 with the reasons for exclusion.

### 3.1 Description of studies included in the review

The included study characteristics are reported in Table 1. Two of the studies reported an intervention,^15^, ^17^ and one looked at the effect of policy change.^16^ One study looked specifically at irregular dental attenders with dental anxiety.^17^ The other two studies included a defined geographical population looking at the effect on the whole population as well as irregular dental attenders.^15^, ^16^ Two studies were set in England,^15^, ^17^ and one in Scotland.^16^ All studies used the broad outcome of attendance for a dental check‐up however measured differently.^15^, ^16^, ^17^

**TABLE 1:** Included study characteristics

Author, year, setting	Aim	Participants	Intervention/Policy Change	Comparator	Outcome	Type of Study
Dailey et al.^17^ 2001, England	To explore, using a predominantly qualitative approach, factors affecting patients’ dental attendance behaviour following referral from a dental anxiety clinic to a general dental practitioner	Adult participants who were irregular dental attenders and who attended a dental anxiety clinic 4 years previously (n = 23)	Dental anxiety clinic	None	Claimed dental attendance behaviour, confirmed with patients’ dental records	Follow‐up observational, qualitative
Anderson and Morgan,^15^ 1992, England	To persuade non‐users or irregular users of dental services to change their behaviour and visit the dental more regularly	Residents of Dudley in the West Midlands of the UK. Population of approximately 300,000. Age group not defined	Promotional campaign for a ‘dental update’	Comparator groups used outside of Dudley for analysis of the awareness of the promotional campaign and number of estimates generated. The comparator groups were, however, partly exposed to the intervention	Number of patients who started courses of treatment. Number of patients requesting a ‘dental update’. Number of dental estimates returned. Awareness of the campaign. Impact of the campaign (qualitative)	Cohort, qualitative
Ikenwilo et al.^16^ 2013, Scotland	To evaluate the effect of the introduction of free NHS dental check‐ups on utilisation in Scotland	Scottish data from the British Household Panel Survey (BHPS)	Introduction of a free dental check‐up	The rest of UK	Number of participants reporting having a check‐up in the previous 12 months	Observational study; Difference in difference analysis

### 3.2 Narrative review of interventions

To summarise the three interventions:

- Dailey et al.^17^ interviewed patients following attendance at a dental anxiety clinic. Treatment at this clinic included behaviour management techniques (including desensitisation, modelling and semi hypnotic suggestion), and dental treatment with nitrous oxide sedation. Patients could also attend an optional dental support group which was described as ‘mutual support in a non‐clinical environment’.
- Anderson and Morgan^15^ reported a generic advertising campaign which aimed to overcome the publics’ perceived barriers to dental care. The promotion also included the option to attend a ‘dental update’, which allowed potential patients to visit a participating dental surgery to meet the dentist and look around the clinic (this did not include dental examination).
- Ikenwilo et al.^16^ looked at the effect of the introduction of a free dental check‐up in Scotland.

A summary of the interventions or policy change mapped to the TDF is provided in Table 2. One study examined the effect of a change in policy to provide free dental check‐ups,^16^ and the other two studies examined interventions.^15^, ^17^ Only one study^15^ described any form of theoretical basis for the intervention that was developed, this being based on previous empirical qualitative findings^23^ indicating that the public image of dental services needed to be improved. None of the studies reported using a theoretical framework for intervention development or directly mapped the interventions or policy change to behaviour change theories. Two studies^15^, ^17^ discussed issues with compliance or execution of the intervention. Attendance at the dental anxiety clinic^17^ resulted in 7 participants of 48 failing to complete the course of treatment, and at four‐year follow‐up only 23 were available for interview, no data were available on the seven participants who failed to complete the treatment. Within a large advertising campaign,^15^ there were multiple problems with execution: posters were displayed across a much larger geographical area than the target population; posters were displayed on buses which moved outside of the target area; leaflets were not delivered due to the delivery company using outdated maps of the area; management of costs with the advertising agency employed led to the campaign not being as widespread as the steering group were initially led to expect. The same study reported issues with compliance of dental practices used in the research leading to two of the five outcome measures being unrecorded. Two studies^15^, ^17^ used co‐interventions, with the dental anxiety clinic also offering the option of a support group which 10 patients attended, and the generic advertising campaign also including a dental professional development programme. The individual impact of these co‐interventions on the outcome measures is unknown. In general, the interventions were poorly reported, with significant omissions in their description and a lack of clear identification of what the intervention entailed.

**TABLE 2:** Summary of intervention/policy change characteristics.

**Ref**	Description of Intervention	Delivery	Providers	Economic Information	Key Findings	TDF
^17^	Attendance at an anxiety clinic and an optional dental support group	Behaviour management techniques included desensitisation, modelling and semi hypnotic suggestion. Nitrous oxide sedation was also available	Anxiety clinic was led by one dentist	None provided	47% became regular dental attenders and had reduced dental anxiety. 53% were irregular or non‐attenders and had higher dental anxiety	Emotion; Reinforcement; Social influences
^15^	Promotional campaign using a celebrity to increase impact over the short term, advertising a free ‘dental update’. This would allow potential patients to visit dental surgeries and ask about treatment, have a look around and meet the dentist (‘window shopping’), this did not include a free dental examination	Dental update logo was produced and displayed on all promotional materials and in prominent places in the participating practices. Advertisements were 3 large roadside hoardings in 30 locations, 4 smaller posters in bus shelters or shopping precincts in 70 locations. A teaser poster was also used in some of the larger hoardings for the 1st week. 13 buses had posters and 5 local newspapers had adverts twice a month. Leaflet also posted to every house Press launch in October with celebrity presence in a large shopping centre	Local advertising agency and local dental practices.	Total cost of campaign approximately £210,000.	The population was aware of the campaign but misinterpreted its message and therefore did not have the desired effect. Only 13% attended for a dental update. There was a reduction in number of dental estimates from the previous year. Other outcome measures failed due to dental practice compliance	Knowledge; Social/professional role and identity
^16^	Free NHS dental check‐up. Patients must register with a dentist and the fee for the check‐up is paid by the Health Boards	No information provided	Government	Average £46 per adult (range £23‐£57)	3–4% increase in NHS dental check‐ups in Scotland compared with the rest of the UK after policy change	Environmental context and resources

### 3.3 Effectiveness of the Interventions

Key findings from the studies are summarised in Table 2. Following attendance at a dental anxiety clinic, almost half of participants became regular dental attenders;^17^ however, only 23 participants out of a total of 41 who received the intervention were included. Only 13% of the population attended for a dental update following a large advertising campaign;^15^ however, there were issues with practice compliance in reporting this outcome; therefore, the true number of attendances could be underestimated. In Scotland, introducing a free dental check‐up resulted in a 3.2% increase in number of dental check‐ups;^16^ however, there was also an increase in the number of patients attending for a private check‐up, and in those who would have been exempt from NHS dental charges before the policy change. A self‐reported outcome measure was also used in this study, which could have introduced reporting bias.

### 3.4 Patient views of interventions

Two studies^15^, ^17^ included a qualitative component to provide patient views on the intervention. Participants attending a dental anxiety clinic who subsequently became regular dental attenders reported behaviour change due to: a transfer of treatment alliance; positive dentist‐patient communication; no resistance formation; positive health beliefs; development of coping mechanisms.^17^ Contrasting viewpoints were reported from those who did not become regular dental attenders. Whilst attending the dental anxiety clinic all participants reported developing a positive relationship with the dentist, which resulted in them being able to receive dental treatment whilst at the clinic; however, this was not always transferred outside of the clinic as participants did not want to receive treatment from another dentist.

A large advertising campaign was used to improve the image of dental services to the general public and promote the opportunity to attend for a free dental update; however, this was largely misinterpreted as ‘visit your dentist’, or ‘there's no need to be frightened of the dentist’.^15^ The dental update was also misunderstood and believed to be the same as a regular dental check‐up. This could partly explain the low uptake of dental updates as approximately half of those interviewed said they would have attended for an update had they been aware. Of those who did attend for a dental update two groups emerged: acknowledgers who reported fear of attending the dentist but felt guilty for not going; opportunists who had toothache at the time of the campaign which prompted them to attend. All were motivated by the opportunity to talk to dentists and being given the chance to discuss any problems or fears, and the fact that dentists agreed to take part in the dental update scheme was considered an indicator that they were friendly and approachable. Interestingly, many had different experiences or could not recall what actually happened at the dental update including if they paid. Those who had no interest in attending a dental update reported no perceived need for seeking dental care, apathy and cost as barriers.

## 4 DISCUSSION

This systematic review highlighted two interventions aiming to increase regular dental attendance in irregular dental attenders and both studies provide areas for consideration in intervention development. Whether attendance at a dental anxiety clinic had a positive behavioural change effect is undeterminable given only half of the patients contacted had changed their attendance behaviour in the longer term, in addition to a large number of patients being lost to follow‐up. However, what was highlighted as having the biggest self‐reported effect on dental anxiety and move into regular dental attendance was the dentist's communication skills and establishing a good dentist‐patient relationship.^17^ It appears that the clinic helped patients receive dental treatment at the time of the intervention, but subsequently transferring care outside of the clinic to a different dentist was a barrier to establishment of routine dental care. This intervention would also only target problem‐orientated attenders who report dental anxiety as a barrier to care seeking, and if this intervention was to be developed further then transfer of care following the intervention would need to be carefully considered in the design process.

A large advertising campaign to promote the image of dental services and offer a free dental update was largely unsuccessful due to public misinterpretation of intervention.^15^ Interestingly, this is the only study identified which reported the theoretical basis for development of the intervention and included dental professionals in the development process. During the intervention design process, all relevant stakeholders should be involved at all stages^24^ to maximise effectiveness and acceptability;^25^ therefore, if patients had been involved as well as dental professionals then the campaign may not have been misinterpreted and been more effective. Additionally, there were multiple problems with execution of this campaign, and had it been delivered as planned, then a larger benefit may have been observed. Finally, the qualitative component to this study highlighted the importance of increasing the awareness of need for dental treatment in any future interventions aimed at irregular dental attenders.

This review also identified one study examining the effect of policy change by introducing a free dental check‐up in Scotland in 2006.^16^ An increase in utilisation of dental check‐ups was noted following this policy change, however, this varied between patient groups, including those accessing private dental care and those who would have been exempt from dental charges prior to the policy change. This therefore raises the question as to whether the observed self‐reported behaviour change was a direct result of the policy change, or whether this indirectly raised awareness of dental services and therefore increased attendance. This study also highlights the wider implications of policy change such as this, including increased workforce requirements and the cost to the NHS and government and raises concerns over sustainability of continued free dental check‐ups. As a result, the authors recommend refining the policy to target more vulnerable groups to maintain an optimal benefit.

As per the *a priori* protocol, a formal risk of bias assessment was not carried out for the studies included in this review as the outcome would not have affected inclusion of the intervention components in a future co‐design process. Whilst systematic reviews with studies indicating a low risk of bias are preferable, when developing interventions it is important to consider and present all relevant studies to stakeholders when discussing the evidence and encouraging blue‐sky thinking.^10^ The studies were critically reviewed as discussed here, and if formal assessment was undertaken would likely show moderate to high risk of bias. Had more studies been available with appropriate design and comparators then risk of bias would have been considered as part of a further effectiveness review to indicate the degree to which the interventions may have been transferable. In addition, grey literature was not included in the search strategy, which may be a potential limitation to the systematic review if any potential interventions have been developed, or policy change trialled, but not published in peer‐reviewed journals.

Across all studies identified the interventions were poorly described, this poses a problem with replication or retrofitting of the interventions in the future and better reporting of intervention components is required. The use of the TIDierR checklist can facilitate reporting of interventions and includes 12 items: brief name; why; what materials; what procedures; who provided; how; where; when and how much; tailoring; modifications; how well (planned); how well (actual).^26^ Due to poor reporting, mapping to the TDF was challenging and there are some limitations to this process with some possible domains potentially being missed. For example, the large advertising campaign^15^ was mapped to knowledge and social/professional role and identity; however, it was unclear from the intervention described as to whether this should have also been mapped to environmental context and resources. The intervention aim was to persuade non‐users or irregular users of dental services to change their behaviour and visit the dentist more regularly by use of a promotional campaign advertising a free ‘dental update’. The dental update was described as allowing potential patients to visit dental surgeries and ask about treatment, have a look around and meet the dentist. On examples of the advertisements used they also suggested that patients could make an appointment for a check‐up whilst at their dental update, which could map to environmental context and resources if this supported appointment making, however, this was not clear in the intervention description and was therefore omitted as a potential TDF domain. Indeed, patients reported different experiences at the dental update in this study, and this could be explained by the poor description to the dentists delivering the intervention.

## 5 CONCLUSION

This systematic review identified a lack of interventions targeted at problem‐orientated dental attendance, however, data within the studies identified highlights the potential importance of considering dentist communication, the dentist‐patient relationship, increasing the awareness of need and the effect and considerations of free dental check‐ups in future intervention development. The same studies also highlighted the importance of a sound evidence base, theoretical frameworks and involvement of relevant stakeholders in intervention development to maximise acceptability and effectiveness.

## CONFLICT OF INTEREST

The authors have no conflicts of interest to declare.

## AUTHOR CONTRIBUTIONS

C Currie contributed to conception, design, data acquisition and interpretation, analysis, drafted and critically revised the manuscript. V Araujo‐Soares and J Durham contributed to conception, design, data acquisition and interpretation, analysis and critically revised the manuscript. SJ Stone and F Beyer contributed to conception, design, data interpretation, and critically revised the manuscript. All authors gave their final approval and agree to be accountable for all aspects of the work.

### PEER REVIEW

The peer review history for this article is available at https://publons.com/publon/10.1111/joor.13244.

## Supporting information

Appendix S1

Appendix S2

Appendix S3

## Data Availability

The data that support the findings of this study are available from the corresponding author upon reasonable request.
