# Supplementary material for: Promoting regular dental attendance in problem‐orientated dental attenders: A systematic review of potential interventions
Source: J Oral Rehabil. 2021 Aug 23;48(10):1183–91. doi: 10.1111/joor.13244 (PMC9292277; doi:10.1111/joor.13244)
Supplement: Supplementary file 1 — Appendix S1 [file JOOR-48-1183-s003.docx]

**Promoting Regular Dental Attendance in Problem Orientated Dental Attenders: A Systematic Review of Potential Interventions**

**Running Title: Interventions for Problem Orientated Attendance**

**Currie CC^1,2^, Araujo-Soares V^3^, Simon SJ^1,2^, Beyer F^4^, Durham J^1,2^**

1. School of Dental Sciences, Newcastle University, Newcastle Upon Tyne, UK
2. Newcastle Upon Tyne Hospitals NHS Trust, Newcastle Upon Tyne, UK
3. Faculty of Behavioural, Management and Social Sciences, University of Twente, The Netherlands
4. Population Health Sciences Institute, Newcastle University, Newcastle Upon Tyne, UK

S1 Appendix: List of databases searched. *Latest issue available

| **Database, platform** | **Search date range or issue date** |
| --- | --- |
| Medline, OVID | Jan 1966 to April 2021 |
| Embase, OVID | Jan 1980 to April 2021 |
| Scopus, Sciverse | Jan 1960 to April 2021 |
| PsycINFO, OVID | Jan 1840 to April 2021 |
| Cochrane Central Register of Controlled Trials (CENTRAL), Wiley Cochrane Library | Issue 7, 2019 |
| Cochrane Database of Systematic Reviews, Wiley Cochrane Library | Issue 7, 2019 |
| Database of Abstracts of Reviews of Effects, Wiley Cochrane Library | Issue 2, 2015* |
| NHS Economic Evaluation Database (NHS EED), Wiley Cochrane Library | Issue 2, 2015* |
