# Supplementary material for: Promoting regular dental attendance in problem‐orientated dental attenders: A systematic review of potential interventions
Source: J Oral Rehabil. 2021 Aug 23;48(10):1183–91. doi: 10.1111/joor.13244 (PMC9292277; doi:10.1111/joor.13244)
Supplement: Supplementary file 3 — Appendix S3 [file JOOR-48-1183-s002.docx]

**Promoting Regular Dental Attendance in Problem Orientated Dental Attenders: A Systematic Review of Potential Interventions**

**Running Title: Interventions for Problem Orientated Attendance**

**Currie CC^1,2^, Araujo-Soares V^3^, Simon SJ^1,2^, Beyer F^4^, Durham J^1,2^**

1. School of Dental Sciences, Newcastle University, Newcastle Upon Tyne, UK
2. Newcastle Upon Tyne Hospitals NHS Trust, Newcastle Upon Tyne, UK
3. Faculty of Behavioural, Management and Social Sciences, University of Twente, The Netherlands
4. Population Health Sciences Institute, Newcastle University, Newcastle Upon Tyne, UK

**Appendix S3**

**Table of Studies Excluded from the Systematic Review**

| **Study** | **Reason for Exclusion** |
| --- | --- |
| Singhal A, Caplan D, Jones M, *et al*. Eliminating medicaid adult dental coverage in California led to increased dental emergency visits and associated costs. *Health Aff (Millwood)*. 34(5):749-756. | Outcome was attendance for urgent dental care at a medical emergency department with no data on attendance for urgent care with dental professionals for comparison over the time period. |
| Moris S, Carty O, Wanyonyi K, Gallagher J. Promoting access to dental care in South London: adult patients’ perspectives. *J Public Health*. 2017;25:601-610. | No interventions actually trialled, instead looking at potential influence of different initiatives to improve uptake of dental care. |
| Cohen LA, Manski RJ, Hooper FJ. Does the elimination of medicaid reimbursement affect the frequency of emergency department dental visits? *J Am Dent Assoc*. 1996;127:605-609. | Outcome was attendance for urgent dental care at a medical emergency department with no data on attendance for urgent care with dental professionals for comparison over the time period. |
| Zittel-Palamara K, Fabiano JA, Davis EL, Waldrop DP, Wysocki JA,  Goldberg LJ. Improving patient retention and access to oral health  care: the CARES program. J Dent Educ. 2005;69:912-918. | Patients with chronic orofacial pain included. |
| Doherty S, Fielder F. The effects of health education on patients’ subsequent dental visits: a practice-based research in health promotions. *Afr Dent J.* 1995;9:9-14. | Outcomes were compliance with treatment given during urgent dental care and return to urgent care for the same condition, did not consider or measure attendance at routine dental care following urgent dental care. |
